# Building access to linked data for program evaluation: lessons and opportunities from the evaluation of a pan-Canadian skills training initiative

**DOI:** 10.23889/ijpds.v9i5.2860

**Published:** 2024-09-18

**Authors:** Max Palamar, Sandra Nkusi

**Affiliations:** 1 Blueprint

In recent years, Canada’s statistical agency (Statistics Canada) has developed sophisticated linkage infrastructure through their Social Data Linkage Environment (SDLE), linking internal data sets held by Statistics Canada and external data held by researchers. While the SDLE presents an opportunity for better measurement of the effectiveness of policies and programs, to date it has not been used for this purpose outside of government, instead largely supporting academic research projects. This session presents a novel approach to program evaluation in the Canadian context, using the SDLE Statistics Canada administrative data alongside independently collected program data from multiple service providers to evaluate the outcomes and effectiveness of a portfolio of skills training programs.

Our approach aims to both rigorously evaluate the long-term outcomes of participants in this portfolio of public skills training programs, and develop a replicable proof-of-concept for using linked data infrastructure in Canada to support program evaluations. Through this initiative, we identify the key design features needed to support this use case for linked data, including study design parameters, necessary datasets and linkage processes, and privacy and data governance policies. Finally, we identify opportunities for future replication of this approach in Canada, including strategies for expedited linkage and analysis of evaluation results that maintain privacy requirements.

We find that using the SDLE infrastructure for program evaluation is both feasible and desirable, however barriers exist to replicating this approach, including sectoral capacity for collecting linkable data, and transposing linkage and confidentiality rules from an academic research to a program evaluation context.

